# Treatment adherence in retinoblastoma: A retro‐prospective cohort study in Ivory Coast and the Democratic Republic of Congo

**DOI:** 10.1002/cnr2.1949

**Published:** 2023-12-26

**Authors:** Robert Mbuli Lukamba, Aléine Nzazi Budiongo, Ben Bondo Monga, Atteby Yao, Pierre Bey, Gabrielle Borasisi Chenge, Laurence Desjardins, François Doz, Albert Tambwe Mwembo, Théophille Amani Kabesha, Oscar Numbi Luboya

**Affiliations:** ^1^ Department of Pediatrics (Pediatric Oncology Unit) University of Lubumbashi Lubumbashi Democratic Republic of the Congo (DRC); ^2^ Department of Pediatrics (Pediatric Oncology Unit) University of Kinshasa Kinshasa Democratic Republic of the Congo (DRC); ^3^ Faculty of Medicine and School of Public Health University of Lubumbashi Lubumbashi Democratic Republic of the Congo (DRC); ^4^ Pediatric Oncology Unit University Teaching Hospital of Treichville Abidjan Ivory Coast; ^5^ Advisor to the President of Institut Curie University of Lorraine and AMCC Paris France; ^6^ Ophthalmology Department University of Lubumbashi Lubumbashi Democratic Republic of the Congo (DRC); ^7^ Ophthalmology Service Institut Curie and AMCC Paris France; ^8^ SIREDO Oncology Center (Care, Innovation, Research in Child Oncology, Adolescent and Young Adult) Institut Curie and University Paris Cité Paris France; ^9^ Bukavu Ophthalmic Clinic Bukavu Official University Bukavu Democratic Republic of the Congo (DRC); ^10^ Department of Pediatrics and School of Public Health University of Lubumbashi Lubumbashi Democratic Republic of the Congo (DRC)

**Keywords:** DRC (Democratic Republic of Congo), Ivory Coast, retinoblastoma, treatment adhesion

## Abstract

**Background:**

In high‐income countries, retinoblastoma is curable in more than 95% of cases, whereas in low‐income countries, mortality remains high, especially when the diagnosis is made late or the treatment is discontinued.

**Aims:**

To determine the factors associated with adherence to the treatment of retinoblastoma in the Ivory Coast and the Democratic Republic of Congo (DRC).

**Methods and Results:**

A retro‐prospective cohort study was carried out. Data were collected from patient folders and follow‐up records of parents.

**Results:**

A total of 175 children with retinoblastoma were registered from January 2013 to December 2015. Seventy‐six children (43%) were 5 years old and above. Care costs were covered by families in 86.9% of cases. Chemotherapy refusal was recorded in 39 cases (22.3%), and enucleation refusal was recorded in 79 cases (45.1%). After 36 months of follow‐up, we recorded 16.6% deaths, 27.4% treatment dropouts, and 18.3% loss to follow‐up after treatment. The commonest cause for enucleation refusal was fear of infirmity, while chemotherapy refusal and absconding treatment were due to financial constraints.

**Conclusion:**

Poor adherence to retinoblastoma management was due to financial constraints, and a lack of knowledge of the disease and its treatment. Family psychosocial support is needed to improve this condition.

## 1 INTRODUCTION

Retinoblastoma is the most common intraocular cancer among childhood cancers. Its global incidence is estimated at 9000 new cases per year, which corresponds to one case in 15 000 births; most in low‐income countries.^1^

Retinoblastoma is cured in more than 95% of cases in high‐income countries.^2^ In an analysis of gross national income of the country versus RB mortality, Chantada et al. reported that survival from RB is 30% in low‐income countries, 60% in lower‐middle‐income countries, 75% in upper‐middle‐income countries, and 95% in high‐income countries.^3^ Recently, Gündüz et al. reported the overall survival rate of 96% in an upper‐middle‐income country.^4^ Thus, in most low‐income countries, the prognosis is still bleak with high mortality reaching 95% in some countries.^5^ The reasons for this disparate and disastrous situation include late diagnosis and treatment, refusal and abandonment of treatment, as well as poor health infrastructures.^5^, ^6^ The abandonment rate of childhood cancer treatment is highest in low‐income countries.^7^ In addition, the retinoblastoma dropout rate in low‐ and middle‐income countries is generally described as between 5% and 40%, respectively.^8^ In most of these countries, the rate of enucleation refusal varies from 1% to 50%,^9^ and the rate of loss of follow‐up after treatment varies from 10% to 75%.^10^, ^11^, ^12^, ^13^, ^14^ This contributes to the high mortality rates of retinoblastoma.

The rates and factors associated with abandonment and refusal of treatment for retinoblastoma are poorly documented in sub‐Saharan Africa.^7^, ^15^ Therefore, it is necessary to identify the factors related to this phenomenon to work to overcome them and draw lessons for other countries in sub‐Saharan Africa. The objectives of this study were to determine: (1) the rate of refusal and abandonment of treatment for retinoblastoma, and (2) the factors associated with adherence to treatment for retinoblastoma in the Ivory Coast and the DRC.

## 2 PATIENTS AND METHODS

### 2.1 Study type and period

This was a retro‐prospective cohort study that used patient data from January 1, 2013, to December 31, 2015.

### 2.2 Study framework

This study was conducted in three cities in the DRC (Lubumbashi, Bukavu, and Kinshasa) and one city in the Ivory Coast (Abidjan). These cities were chosen due to the participation of their pediatric oncology units as well as the ophthalmological center of Bukavu in a research collaboration within the framework of a program for early diagnosis and access to treatment of retinoblastoma in sub‐Saharan Africa. The program was initiated by the Global Alliance Against Cancer (AMCC) in collaboration with the Franco–African Pediatric Oncology Group (GFAOP) and the Institut Curie, supported by the SANOFI ESPOIR foundation as part of its My Child Matters program.^16^

### 2.3 Population and sampling

The study involved children with retinoblastoma aged 0 to 17 years. Children whose records did not contain essential information (sex, age, laterality, acceptance of treatment, date of the last news or death, or end of treatment) were excluded from the analysis.

The children were included in the study after obtaining written or oral informed consent from their parents (caregivers), in accordance with the local recommendations and the guidelines of the GFAOP.^17^ This study received approval from the ethics committees of the University of Lubumbashi and the Felix Houphouët Boigny University of the Ivory Coast.

### 2.4 Data collection

Data collection was performed from August 16 to December 31, 2015. The data were collected from patient files and interviews of the parents of former and new patients according to a pre‐established questionnaire given to a resource person in each of the centers. Training of investigators was provided at the various sites. The survey sheet had two sections. In the first section, data were collected only once on admission; the collection of data stopped in this section for cases with treatment refusal (chemotherapy and surgery).Chemotherapy regimen used was:

- 2 neoadjuvant CE (carboplatin–etoposide) cycles for advanced intraocular retinoblastoma and alternate CE and CO (cyclophosphamide–oncovin) for adjuvant chemotherapy if indicated.
- CEV (cyclophosphamide–vincristin–etoposide) for orbitary or bilateral retinoblastoma.
  Enucleation was indicated for all intraocular and intraorbitary retinoblastoma, and also in palliative traitment of metastatic cases. The conservative treatment was not avaiblable at all our retinoblastoma treatment centers.

In the second section, the data was collected several times during the treatment visits while noting all the events that occurred before each cycle of chemotherapy. It was filled out until the end of treatment, or the last visit during the study period, the latest action (if treatment was abandoned), or until death if it had occurred during the study period. For former cases, the files were consulted, and additional information was obtained by contacting the parents by telephone unless they could be met during their follow‐up appointment. For new cases (referred during the study period or treatment), the forms were completed during the clinical examination and supplemented by the parents' interviews.

The following variables were collected: sex, age, nationality, siblings, residence, date of diagnosis, evolutionary form (intraocular or extraocular), acceptance of chemotherapy, acceptance of enucleation, regularity of treatment, reasons for refusal or abandonment of treatment, marital status of the parents, level of education of the parents, the profession of the parents, coverage of care, date of the latest visit, and child's condition at the latest visit. ICRB groups were not described because of precarious conditions of work: most of fundus copies were performed without general anesthesia.

Coverage of care was considered “unsecured” if the care was financed by the family, and “secured” if it was financed by a social solidarity system (mutual fund, social security fund, employer, etc.). We considered the refusal of treatment as the decision of the parents to have no treatment. Abandonment of treatment was defined as the refusal to continue a curative treatment already started, with the patient missing the appointment at least 4 weeks after the scheduled appointment date. Treatment adherence was defined as acceptance of the proposed treatment and following it to the end.

### 2.5 Data analysis

Data entry was performed using Excel 2010 software (Microsoft Excel Corp., USA), and data were analyzed with SPSS version 24 for Windows (IBM, Armonk, NY, USA). A comparison of proportions, medians, and means was carried out using Chi‐square, Mann–Whitney, Wilcoxon, and Student's *t*‐tests. The Kaplan–Meier method was used to estimate the survival curve, the incidence of treatment drop‐out, and the loss of follow‐up. Logistic regression looked for independent determinants of treatment refusal; .05 *p*‐value variables were selected for multivariable analysis after univariate analysis Cox regression looked for independent predictors of mortality, treatment dropout, and loss of follow‐up. A value of *p* < .05 was considered the threshold of statistical significance.

## 3 RESULTS

### 3.1 Sociodemographic and clinical characteristics of patients

A total of 176 consecutive patients with retinoblastoma were registered. One incomplete file was excluded from the analysis; thus, 175 children were evaluated. The ratio between the sexes was 1.03 (89 boys and 86 girls). The median age at diagnosis was 33 months (range: 2–108; IQR [Interquartile range]: 23–45). Urban residence was reported in 118 cases (67.4%), and the median distance between the usual residence and the care center was 30 km (range: 1–1000; IQR: 10–116). The parents were married in 83.3% of cases (105/126). Moreover, care was financed by families in 86.9% of cases (126/145), with social solidarity systems (mutual insurance company, social security fund, employer, etc.) being less frequent.

The median number of siblings was 4 children (range: 2–12; IQR: 2–5). The median rank of the sick child among his/her siblings was 3 (range: 1–12; IQR: 2–4). Unilateral affection occurred in 156 cases of retinoblastoma (89.1%), and bilateral affection occurred in 19 cases (10.9%). Regarding staging, retinoblastoma was intraocular in 45.7% of cases (80/175) and extraocular in 54.3% (95/175).

### 3.2 Adherence to treatment

We have not found any relationship between many variables like the number of siblings and the % of children agreeing to take the chemo and/or enucleation treatment. Thus, their association with treatment adherence has not been described.

### 3.3 Frequency and causes of chemotherapy refusal

Refusal of chemotherapy was recorded in 39 cases (22.3%). Financial difficulties were the first cause (in 61.5% of cases), followed by the fear of side effects (10.3%) and negative beliefs (10.3%).

### 3.4 Determinants of chemotherapy acceptability

Chemotherapy was accepted much more often when the parents were married and when the care was financed by a social solidarity system, mainly a company social fund or a mutual insurance company, odds ratio (OR) = 3.95, 95% confidence interval (CI 95%) = 1.13–8.47, *p* = .027; (Table 1).

**TABLE 1:** Determinants of chemotherapy acceptability according to the multivariate logistic regression model.

Variables	Adjusted OR (CI 95%)	*p*‐value
Marital status		
Other (1)	1	
Divorced	1.29 (0.56–1.45)	.130
Married	2.53 (1.06–4.00)	**.019** ^a^
Center		
Bukavu	1	
Lubumbashi	0.91 (0.17–4.82)	.908
Abidjan	0.20 (0.03–2.34)	.200
Kinshasa	1.42 (0.29–7.09)	.669
Coverage of care (2)		
Unsecured	1	
Secure	3.95 (1.13–8.47)	**.027** ^a^
Father's profession		
Farmer/craftsman	1	
Worker	1.34 (0.13–3.96)	.808
Liberal	1.10 (0.25–1.41)	.998
Manager‐agent	1.75 (0.50–2.40)	.839
First health professional consulted		
Traditional healer	1	
General practitioner	1.13 (0.04–2.14)	.678
Ophthalmologist	1.43 (0.41–2.58)	.078
Pediatrician	3.30 (1.13–6.13)	**.022**
Nurse	6.25 (3.12–13.51)	**.007** ^a^

*Note*: (1) Other: deceased, cohabiting, engaged, separated, and widower. (2) Coverage of care was unsecured if the care was financed by the family, and secure if it was financed by a social solidarity system (mutual fund, social security fund, employer, etc.).

Abbreviations: CI, confidence interval; OR, odds ratio.

^a^ Chemotherapy was much more accepted when the parents were married, when the care was financed by a social solidarity system and when the first healthcare professional consulted was a nurse or a pediatrician.

### 3.5 Frequency and causes of enucleation refusal

In total, refusal of enucleation occurred in 79 cases (45.1%); neverless, after chemotherapy enucleation was recommended to 115 patients from whom 82 have accepted.

Fear of infirmity was the main cause (in 37.4% of cases), followed by financial difficulties (20.3%). Fear of stigmatization was mentioned in 13.9% of cases.

### 3.6 Determinants of enucleation acceptability

Results from the multivariate analysis showed that the main determinants of enucleation refusal were (Table 2):

- Laterality; enucleation was three times more accepted when retinoblastoma was unilateral than when it was bilateral, OR = 3.50; CI 95% = 1.17–6.19; *p* = .035
- The profession of the father; enucleation was more accepted when the father was an agent or manager of public service or a company; OR = 2.79; CI 95% = 1.81–8.81, *p* = .023.

**TABLE 2:** Determinants of acceptance of enucleation according to the multivariate logistic regression model.

Variables	Adjusted OR (CI 95%)	*p*‐value
Mother's education level		
None/elementary	1	
High school	1.27 (0.06–2.27)	.712
University	1.67 (1.81–5.56)	**.018** ^a^
Location of retinoblastoma		
Bilateral	1	
Unilateral	3.50 (1.17–6.19)	**.035** ^a^
Fathers' profession		
Farmer/craftsman	1	
Worker	1.19 (0.53–2.57)	.131
Liberal	1.51 (1.09–7.82)	.053
Manager‐agent (1)	2.79 (1.81–8.81)	**.023** ^a^
Mother's occupation		
Farmer/craftsman	1	
Liberal	1.18 (0.19–7.42)	.863
Worker	1.50 (0.20–11.26)	.692

*Note*: (1) Executive of a company, public service, or office worker.

Abbreviations: CI, confidence interval; OR, odds ratio.

^a^ Enucleation was three times more accepted when the retinoblastoma was unilateral and when the father was an agent or manager of the public service or a company. It was slightly more accepted when the mother was college‐educated.

### 3.7 Frequency and causes of treatment discontinuation

**FIGURE 1:**
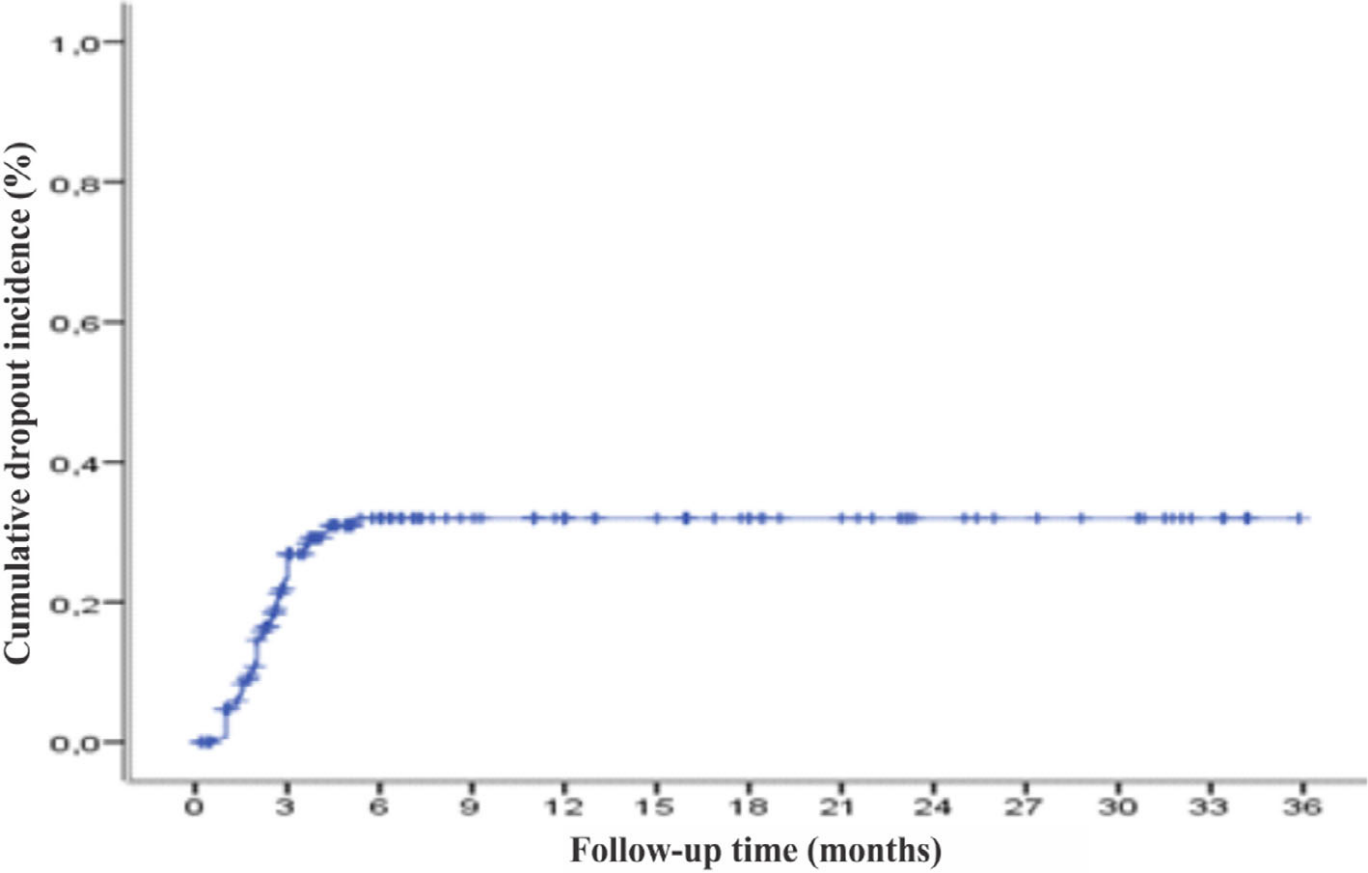
Cumulative dropout incidence in the study population. The treatment was abandoned in 48 cases (27.4%). Most cases of discontinuation of treatment occurred during the first 6 months.

Treatment was abandoned in 48 cases (27.4%), and financial difficulties (in 54.2% of cases) were the main cause of abandonment. Fear of side effects (effects of enucleation and chemotherapy) was mentioned in 12.5% of cases and fear of ineffectiveness in 10.4% of cases. The majority of treatment dropouts occurred during the first 6 months (Figure 1).

### 3.8 Predictors of treatment dropout

The main predictors of treatment dropout were (Table 3):

- The method of financing care; there were three times more dropouts from treatment when care was paid for by the family than when it was financed by a social solidarity system.
- The treatment carried out at the beginning; when the initial treatment was chemotherapy, there were eight times more dropouts from treatment than when it was enucleation.

**TABLE 3:** Predictors of dropout according to the Cox proportional hazards model.

Risk factors	Person‐month (P‐M)	*n*	Incidence of abandonment for 100 P‐M	HR adjusted (95% CI)	*p*‐value
Secure coverage of care (1)					
Yes	40	10	11.1	1	
No	409	38	29.9	2.75 (1.79–4.84)	**.016** ^a^
Consultation of traditional healer					
No	154	37	11.6	1	
Yes	96	11	24.2	2.73 (1.28–5.82)	**.009** ^a^
First treatment					
Enucleation	11	4	7.6	1	
Chemotherapy	373	44	23.5	7.75 (2.59–23.21)	**<.001** ^a^

*Note*: (1) Coverage of care was unsecured if the care was financed by the family, and secure if it was financed by a social solidarity system (mutual fund, social security fund, employer, etc).

Abbreviations: CI, confidence interval; HR, hazards ratio.

^a^ There were three times more people abandoning treatment when the care was paid for by the family than when it was financed by a social solidarity system, three times more abandonment when the family consulted a traditional healer, and eight times more treatment dropouts when the initial treatment was chemotherapy than when it was enucleation.

### 3.9 Frequency of loss to follow‐up

There were 32 cases (18.3%) lost to follow‐up after treatment.

### 3.10 Loss to follow‐up predictors

The independent predictors of loss to follow‐up were (Table 4) as follows:

- The method of financing care; there were three times more people lost to follow‐up when care was paid for by the family than when it was financed by a social solidarity system.
- The treatment carried out at the beginning; when the initial treatment was chemotherapy, there was three times more loss to follow‐up than when it was enucleation.

**TABLE 4:** Predictors of loss to follow‐up according to the Cox proportional hazards model.

Risk factors	Person‐month (P‐M)	*n*	Incidence of loss to follow‐up for 100 P‐M	HR adjusted (95% CI)	*p*‐value
Secure coverage of care (1)					
Yes	8	2	11.1	1	
No	380	30	35.2	2.74 (1.78–3.90)	**.019** ^a^
Consultation of traditional healer					
No	48	15	8.9	1	
Yes	107	17	17.5	1.79 (0.86–3.74)	**.122**
First treatment					
Enucleation	28	9	8.6	1	
Chemotherapy	157	23	19.0	2.85 (1.61–10.94)	**.017** ^a^

*Note*: (1) Coverage of care was unsecured if the care was financed by the family, and secure if it was financed by a social solidarity system (mutual fund, social security fund, employer, etc).

Abbreviations: CI, confidence interval; HR, hazards ratio.

^a^ There were three times more people lost to follow‐up when care was paid for by the family than when it was financed by a social solidarity system; three times more lost to follow‐up when the initial treatment was chemotherapy than when it was enucleation.

### 3.11 Patient survival

**FIGURE 2:**
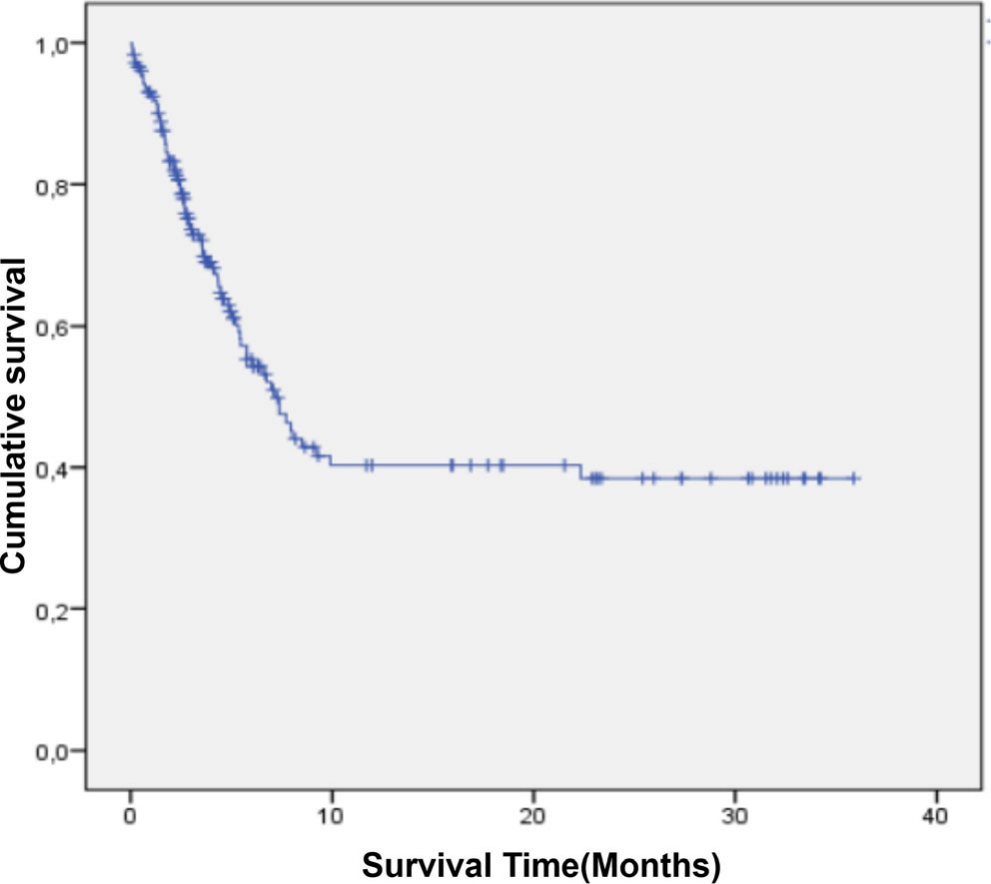
Overall survival. The probability of survival was 63.4% (95% CI: 56.3–70.5) at 6 months, 56.6% (95% CI: 56.3–70.5) at 12 months, and 56% (95% CI: 48.7–63.4) at 36 months.

**FIGURE 3:**
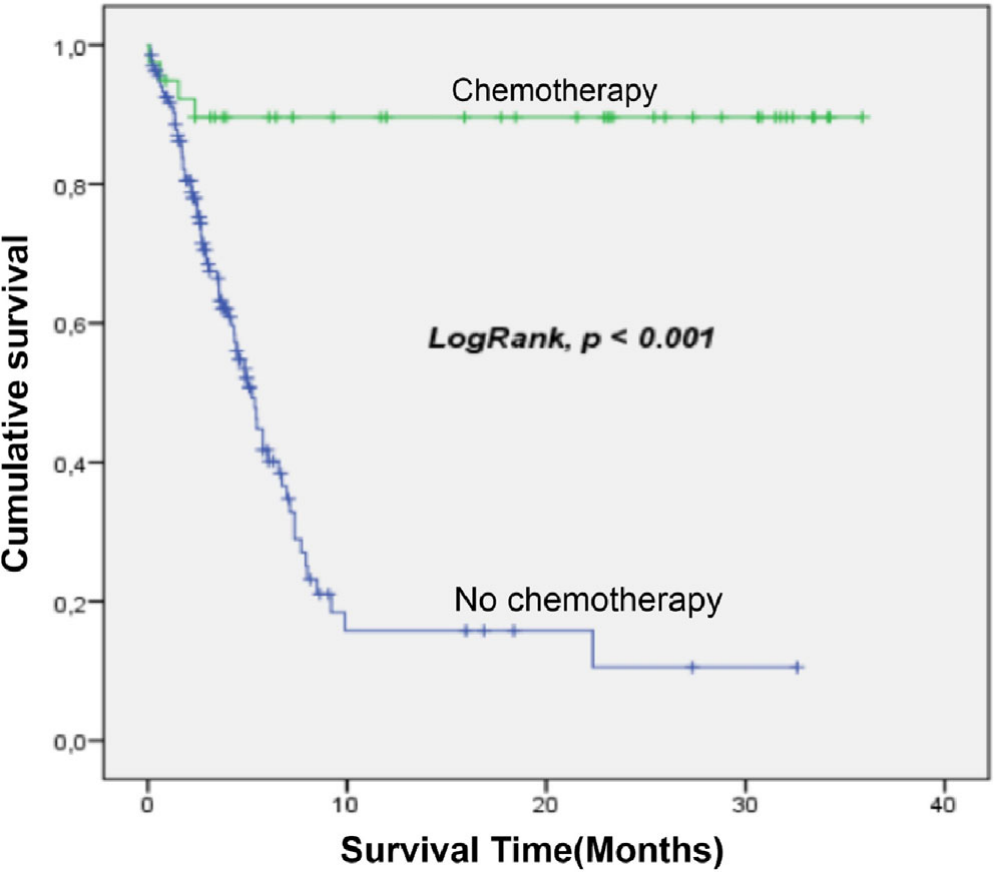
Survival depending to chemotherapy. There was significatively more survival among children whom chemotherapy was fully administrated.

**FIGURE 4:**
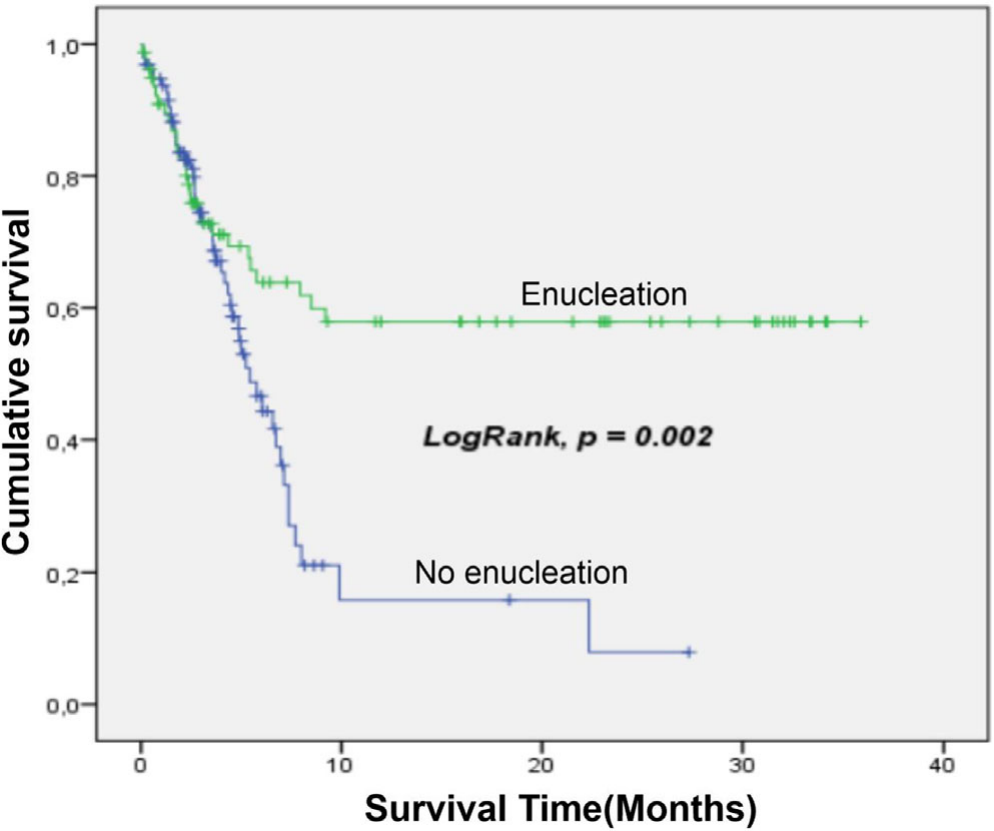
Survival depending to enucleation. There was significatively more survival among children whom enucleation was performed.

Death occurred in 29 cases out of 95 (30.5%), treatment discontinuation patients were excluded from the analysis. The probability of survival was 56% (CI 95%: 48.7–63.4) at 36 months. The median survival time was 3.7 months (interquartile range [IQR]: 2.1–7.3). The critical period corresponds to the first 6 months with a death rate of 36.6%. The main predictors of mortality were the progressive form of retinoblastoma and the refusal of treatment (Table 5, Figures 2, 3 and 4).

**TABLE 5:** Predictors of mortality according to the Cox proportional hazards model.

Variables	Adjusted HR (95% CI)	*p*‐value
Age		
≥5 ans	1	
<5 ans	1.40 (0.59–3.33)	.44
Stage		
Intraocular	1	
Extraocular	8.21 (1.89–35.57)	**.005** ^a^
Chemotherapy acceptance		
Yes	1	
No	6.38 (1.39–9.28)	**.017** ^a^
Enucleation acceptance		
Yes	1	
No	2.23 (0.99–5.00)	.052
Consultation of a traditional healer		
No	1	
Yes	1.82 (0.21–2.79)	.232

Abbreviations: CI, confidence interval; HR, hazards ratio.

^a^ Mortality was higher when there were extraocular forms and treatment refusal.

## 4 DISCUSSION

Adherence to treatment for retinoblastoma was low in the Ivory Coast and the DRC with 22.3% refusal of chemotherapy, 45.1% refusal of enucleation, 27.4% abandonment of treatment, and 18.3% loss to follow‐up after treatment.

The global rate of refusal and abandonment of treatment for childhood cancers is recorded; however, results are distributed unevenly with less than 10% of cases in high‐income countries.^5^ In contrast, it can reach up to more than 90% in low‐income countries where the refusal of enucleation can go up to 100% of cases.^9^ Ye described 35.8% of chemotherapy refusal cases in China,^18^ while Malliptana reported 0.8% of refusal cases in a series in Canada.^19^

There has also been a regional disparity in the rate of loss of follow‐up worldwide: 13% to 16% in some countries with limited resources,^18^, ^20^ 18% to 36% in some African countries,^21^, ^22^ and lower rates in developed European countries such as Italy where 5.6% of patients were lost to follow‐up during treatment for retinoblastoma by intra‐arterial injection of melphalan,^23^

Our chemotherapy refusal rate matches other low‐ and middle‐income countries. The financial difficulties, which are the main causes of the refusal of chemotherapy in our environment, could reflect the inaccessibility to medical care in most French‐speaking sub‐Saharan African countries,^24^, ^25^ Enucleation refusal rates like ours have been described in sub‐Saharan Africa, where a large proportion of deaths related to enucleation refusal have been noted in the absence of care improvement programs,^26^, ^27^ The most frequent causes are belief in traditional treatments or the fatality of cancer as well as fear of the esthetic outcomes resulting from surgery, and fear of stigmatization.^9^, ^28^ Despite the similarity to other low or middle‐income countries, our results had the distinction of having financial difficulties as the second cause after the fear of infirmity.

Bilaterality was a factor that influenced the refusal of enucleation in our study, which was similar to the findings of a series in India,^29^ In our countries where conservative treatment is not well developed yet, the fear of bilateral enucleation could explain this parental attitude.

In this study, which is the first of its kind in French‐speaking sub‐Saharan Africa, we found that the absence of secure management of medical care, the unmarried status of parents, the low level of education, and the low qualification of the parents professions were determinants and predictors of the refusal and abandonment of treatment.

Financial difficulties are the main causes of abandonment of treatment described in other countries, particularly in Asia,^29^, ^30^, ^31^, ^32^ Other causes include the fear of enucleation,^29^, ^30^ and difficulties related to travel for families who live far from care centers,^31^, ^32^ In Africa, financial difficulties and the unavailability of drugs are reported as reasons for abandoning treatment^5^ due to poor funding through social security and health insurance systems. In principle, we reported similar causes, and fear of side effects (including fear of the consequences of enucleation) was among the causes of discontinuation of treatment. Moreover, difficulties in traveling to the specialized center and the unavailability of medication may be the consequences of financial difficulties.

Consultation with the traditional healer is a particularity that we found to be a predictor of the abandonment of the treatment. This could be explained by the fact that those who resort first to this category of health professionals believe less in the effectiveness of modern treatment. However, we do not have a particular explanation for the influence of consulting other health professionals (nurses, general practitioners, and pediatricians) on the refusal or abandonment of treatment.

Furthermore, we had many more dropouts when the treatment started with chemotherapy: this may be due to the fact that the parents dropped out of the treatment at the time of enucleation; whereas the opposite was observed in Latin America, where neo‐adjuvant chemotherapy made it possible to accept enucleation in a certain number of children,^5^, ^33^ Perhaps our families were less prepared for enucleation, even when chemotherapy treatment was started.

Ultimately, the financial difficulties of the parents and their understanding of the disease and its treatment are major determinants of refusal and abandonment of treatment. Psychosocial and financial support programs for families (with better communication on the ocular prosthesis, the prognosis, etc.) can improve adherence to treatment for childhood cancers and survival, as has been proven elsewhere,^34^, ^35^

Survival linked to retinoblastoma varies greatly according to geographic location. In developed countries, survival is more than 97%,^36^ with preservation of vision in at least one eye reaching 90% of cases.^2^ However, in several African and Asian countries, the mortality rate varies between 40% and 70%,^35^ Socioeconomic factors as well as the refusal and abandonment of treatment contribute to this high mortality,^5^, ^37^ The 56% 3‐year survival rate found in our study is consistent with rates found in sub‐Saharan African countries. Most of the patients who dropped out of treatment did so within the first 6 months. This matches the results of a study in East Africa where retinoblastoma‐related survival was difficult to estimate because most children were lost to follow‐up during the first year of treatment,^38^

Advanced forms of retinoblastoma are generally associated with low socioeconomic status^38^ which negatively influences (with refusal or abandonment of treatment) survival,^37^ as observed in our study.

The median age at diagnosis is 14 months in high‐income countries and 30.5 months in low‐income countries,^39^ Age was slightly higher in our study as was the number of cases with advanced forms of retinoblastoma. This may be explained by the association of advanced age with advanced forms of retinoblastoma.^39^

The sex ratio was approximately 1 in our study. Other studies reported that there is often a slight male predominance without any impact on the evolutionary forms or survival,^39^, ^40^ Nevertheless, studies from India occasionally report a predominance of the refusal or abandonment of treatment in females, which probably follows the cultural discrimination in favor of boys in the attention granted to medical care by parents.^29^

Siblings are not described as a factor affecting treatment adherence. However, the number of people per room in the house is involved in the calculation of certain indices of socioeconomic level, which influence treatment,^41^ We can estimate that a large number of children per family is associated with a low socioeconomic level which would lead to difficulties in accessing medical care.

One limitation of our work was that we were unable to find all the information we wanted in the retrospective files despite the effort to complete them by telephone. However, this study has identified the main factors influencing adherence to treatment in our settings. This information could be better supplemented by a subsequent qualitative study.

## 5 CONCLUSION

Adherence to retinoblastoma treatment was low in the Ivory Coast and the DRC. Refusal and abandonment of treatment for retinoblastoma were mainly linked to unsecured coverage of care, the socioeconomic conditions of the parents, and their apprehension of the effects of treatment, in particular the fear of the effects of enucleation. In 2018, the World Health Organization launched a global initiative to combat childhood cancer,^42^ the aim of which is to improve the prognosis of this pathology in low‐ and middle‐income countries. To achieve these objectives, technical support for public health actions by governments is gradually being carried out. Six cancers have been selected as priorities, including retinoblastoma. We hope that the prognosis of retinoblastoma can thus be improved in our countries, through better‐organized psychosocial support for parents.

## AUTHOR CONTRIBUTIONS

**Robert Mbuli Lukamba:** Conceptualization (lead); data curation (lead); formal analysis (equal); funding acquisition (lead); investigation (lead); methodology (equal); project administration (lead); resources (equal); software (equal); validation (equal); visualization (lead); writing – original draft (lead); writing – review and editing (lead). **Aléine Nzazi Budiongo:** Conceptualization (equal); data curation (equal); formal analysis (equal); investigation (equal); methodology (equal); project administration (equal); resources (equal); validation (equal); writing – review and editing (equal). **Ben Bondo Monga:** Conceptualization (equal); formal analysis (lead); methodology (lead); software (lead); supervision (lead); validation (equal); writing – review and editing (equal). **Atteby Yao:** Data curation (equal); formal analysis (equal); investigation (equal); methodology (equal); project administration (equal); resources (equal); validation (equal); writing – review and editing (equal). **Pierre Bey:** Formal analysis (equal); funding acquisition (equal); methodology (equal); project administration (equal); supervision (equal); validation (equal); writing – review and editing (equal). **Gabrielle Borasisi Chenge:** Formal analysis (equal); methodology (equal); supervision (equal); validation (equal); writing – review and editing (equal). **Laurence Desjardins:** Formal analysis (equal); methodology (equal); supervision (equal); validation (equal); writing – review and editing (equal). **François Doz:** Conceptualization (equal); formal analysis (lead); methodology (lead); project administration (equal); software (equal); supervision (lead); validation (lead); visualization (equal); writing – review and editing (lead). **Albert Tambwe Mwembo:** Formal analysis (lead); methodology (equal); project administration (equal); software (equal); supervision (lead); validation (lead); visualization (equal); writing – review and editing (lead). **Théophille Amani Kabesha:** Data curation (equal); formal analysis (equal); investigation (equal); methodology (equal); project administration (equal); resources (equal); validation (equal); writing – review and editing (equal). **Oscar Numbi Luboya:** Formal analysis (equal); methodology (equal); project administration (equal); supervision (lead); validation (equal); visualization (equal); writing – review and editing (lead).

## FUNDING INFORMATION

This research received no external funding.

## CONFLICT OF INTEREST STATEMENT

The authors have no conflicts of interest to declare.

## Data Availability

The data that support the findings of this study are available from the corresponding author upon reasonable request.
